# APOL1 Modulates Renin–Angiotensin System

**DOI:** 10.3390/biom14121575

**Published:** 2024-12-10

**Authors:** Vinod Kumar, Prabhjot Kaur, Kameshwar Ayasolla, Alok Jha, Amen Wiqas, Himanshu Vashistha, Moin A. Saleem, Waldemar Popik, Ashwani Malhotra, Christoph A. Gebeshuber, Karl Skorecki, Pravin C. Singhal

**Affiliations:** 1 Department of Medicine and Feinstein Institute for Medical Research, Zucker School of Medicine, Hempstead, NY 11549, USA; vinsh777@gmail.com (V.K.); prabhjotkaur311090@gmail.com (P.K.); kayasol1@hfhs.org (K.A.); ajha1@northwell.edu (A.J.); wiqasaam@gmail.com (A.W.); hvashist@northwell.edu (H.V.); malhotas@gmail.com (A.M.); 2 Department of Nephrology and Dermatology, Postgraduate Institute for Medical Research, Chandigarh 160012, India; 3 Department of Pediatrics, Bristol School of Medicine, University of Bristol, Bristol BS8 1UD, UK; m.saleem@bristol.ac.uk; 4 Center for AIDS Health Disparity, Meharry Medical College, Nashville, TN 37208, USA; wpopik@mmc.edu; 5 Clinical Institute for Pathology, Medical University of Vienna, 1090 Vienna, Austria; christoph.gebeshuber@meuniwien.a.at; 6 Azrieli Faculty of Medicine, Bar-Ilan University, Safed 1311502, Israel; karl.skorecki@biu.ac.il

**Keywords:** APOL1, podocyte, renin-angiotensin system, miR193a, VDR, WT1, BASP1

## Abstract

Patients carrying APOL1 risk alleles (G1 and G2) have a higher risk of developing Focal Segmental Glomerulosclerosis (FSGS); we hypothesized that escalated levels of miR193a contribute to kidney injury by activating renin–angiotensin system (RAS) in the APOL1 milieus. Differentiated podocytes (DPDs) stably expressing vector (V/DPD), G0 (G0/DPDs), G1 (G1/DPDs), and G2 (G2/DPDs) were evaluated for renin, Vitamin D receptor (VDR), and podocyte molecular markers (PDMMs, including WT1, Podocalyxin, Nephrin, and Cluster of Differentiation [CD]2 associated protein [AP]). G0/DPDs displayed attenuated renin but an enhanced expression of VDR and Wilms Tumor [WT]1, including other PDMMs; in contrast, G1/DPDs and G2/DPDs exhibited enhanced expression of renin but decreased expression of VDR and WT1, as well as other PDMMs (at both the protein and mRNA levels). G1/DPDs and G2/DPDs also showed increased mRNA expression for Angiotensinogen and Angiotensin II Type 1 (AT1R) and 2 (AT2R) receptors. Protein concentrations of Brain Acid-Soluble Protein [BASP]1, Enhancer of Zeste Homolog [EZH]2, Histone Deacetylase [HDAC]1, and Histone 3 Lysine27 trimethylated [H3K27me3] in WT1-IP (immunoprecipitated proteins with WT1 antibody) fractions were significantly higher in G0/DPDs vs. G1/DPD and G2/DPDs. Moreover, DPD-silenced BASP1 displayed an increased expression of renin. Notably, VDR agonist-treated DPDs showed escalated levels of VDR and a higher expression of PDMMs, but an attenuated expression of renin. Human Embryonic Kidney (HEK) cells transfected with increasing APOL1(G0) plasmid concentrations showed a corresponding reduction in renin mRNA expression. Bioinformatics studies predicted the miR193a target sites in the VDR 3′UTR (untranslated region), and the luciferase assay confirmed the predicted sites. As expected, podocytes transfected with miR193a plasmid displayed a reduced VDR and an enhanced expression of renin. Renal cortical section immunolabeling in miR193a transgenic (Tr) mice showed renin-expressing podocytes. Kidney tissue extracts from miR193aTr mice also showed reduced expression of VDR and PDMMs, but enhanced expression of Renin. Blood Ang II levels were higher in miR193aTr, APOLG1, and APOL1G1/G2 mice when compared to control mice. Based on these findings, miR193a regulates the activation of RAS and podocyte molecular markers through modulation of VDR and WT1 in the APOL1 milieu.

## 1. Introduction

Americans with recent Sub-Saharan African ancestry develop chronic kidney diseases at a higher rate when compared to Americans without such ancestry [1,2]. This disparity has been attributed to two sets of coding variants in the APOL1 gene. The APOL1 G1 variant encodes two amino acid substitutions (Serine to Glycine [S to G] at 342 and Isoleucine [I] to Methionine [M] at 384) near the C terminus of the protein, whereas the APOL1 G2 gene product has a two-amino-acid deletion (Asparagine [N] at 388 and Tyrosine [Y] at 389) [1,2]. The increased risk in individuals with genotypes comprising two APOL1 risk alleles (compound heterozygous—G1/G2 or homozygous for the G1 or G2 alleles—G1/G1 or G2/G2) is approximately fourfold to sevenfold for hypertension-associated end-stage renal disease, 17-fold for Focal Segmental Glomerular Sclerosis (FSGS), and 29- to 89-fold for HIV-associated nephropathy when compared to individuals with no risk allele (G0/G0), as well as more significant than that for individuals with only a single risk allele (G0/G1 or G0/G2) [1,2,3,4,5]. Pharmacologic inhibition or blockade of the renin-angiotensin system (RAS) is part of the standard management to slow the progression of many forms of chronic kidney disease (CKD) [6]. Activation of the local RAS in kidney cells also causes kidney cell injury and contributes to the progression of CKD through mechanisms that have been extensively investigated [7,8]. However, the interaction of APOL1 and its risk alleles in activating the RAS has yet to be investigated.

The profile of the systemic RAS in hypertensive African Americans (AAs) has been reported to display a relatively greater degree of activation of the intrarenal compared to systemic RAS than is the case for hypertensive patients of predominantly European ancestry, like what has been reported for diabetic patients [9,10,11,12,13]. Therefore, we sought to determine whether the APOL1-miR193a axis plays a role in activating the RAS in podocytes (a kidney cell with the cardinal role in maintaining the glomerular filtration barrier) under normal physiological and pathological states. We hypothesized that APOL1G0 carries the potential to down-regulate podocyte renin expression, whereas APOL1 risk alleles lack this property and would instead be permissive to escalation of the expression of renin and associated downstream signaling.

We have earlier reported that the expression of APOL1 wild-type (non-risk allele, G0) down-regulates the expression of miR193a and prevents podocyte injury in adverse milieus [14]. In contrast, APOL1 risk alleles enhance the expression of miR193a and induce podocyte injury [15,16]. miR193a has been reported to inversely regulate the expression of WT1 [17,18,19]; since WT1 is a master transcriptional regulator of podocyte gene expression, a reduction in its expression and activity in a high miR193a milieu would be detrimental to podocyte health. Moreover, WT1 is a repressor of renin transcription [20]. Therefore, its reduction would be expected to escalate the generation of renin. Multiple complex partners determine WT1-mediated downstream signaling [21]. They determine WT1 signaling specificity by transcriptional repression or activation. Since we were analyzing the RAS in podocytes, we look to identify a WT1 co-repressor that participates in other WT1-associated transcription of podocyte genes. WT1 contains a suppression domain at its N-terminus that inhibits the function of the transcriptional domain [22]. Brain Acid-Soluble Protein 1 (BASP1) is a WT1 transcriptional co-repressor that mediates this inhibition [23]. BASP1 is present during embryogenesis, and like WT1, BASP1 persists in the podocyte cells of the adult kidney. In the present study, we examined the role of WT1-BASP1 co-repressors in the modulation of renin generation in the podocytes.

MicroRNA (miR) 193a plays a critical role in the development of podocytes and parietal epithelial cells (PECs) during embryogenesis [18]. Both glomerular parietal epithelial cells (PECs) and podocytes originate from the same mesenchymal cells. However, the expression of miR193a-5p determines whether cells would display a PEC or podocyte phenotype. In in vitro studies, undifferentiated podocytes displayed enhanced expression of miR193a but lacked podocyte markers, whereas differentiated podocytes exhibited decreased expression of miR193a but a robust expression of podocyte molecular markers [14]. We demonstrated earlier that miR193a bound to mRNA of the APOL1 3’ UTR region and decreased the expression of APOL1 mRNA [19]. However, the downregulation of miR193a also enhanced the expression of APOL1, which participated in the maintenance of the podocyte molecular phenotype [18,19]. Interestingly, an increase in miR193a expression led to an upregulation of the expression of PEC markers, whereas a decreased expression of miR193a enhanced the molecular markers of podocytes [19].

miR193a also induces oxidative stress and promotes apoptosis in podocytes [17]; this effect of miR193a has been implicated in the development and progression of FSGS in humans and experimental animal models [17]. Similarly, the RAS induces oxidative stress and promotes podocyte apoptosis [24,25]. Therefore, miR193a may contribute to podocyte injury partly by activating the RAS in podocytes.

Liganded and unliganded Vitamin D receptors (VDRs) negatively regulate renin expression in podocytes [26,27]. We asked whether miR193a enhances renin expression in podocytes by reducing VDR. Interestingly, WT1 has been reported to enhance the transcription of VDR in HEK cells [28]; since miR193a negatively regulates the transcription of WT1 [17,18], it could upregulate renin expression through a reduction in VDR via modulation of the transcription of WT1.

In the present study, we evaluated the effect of miR193a expression on the renal and podocyte activation of the RAS for both APOL1 risk and non-risk variants in kidneys in general and podocytes. To confirm the role of the APOL1-miR193a axis on the modulation of renin expression, we examined the expression levels of the Vitamin D receptor and WT1-BASP1 repressor complexes, which play regulatory roles in the expression of renin.

## 2. Methods

**Podocyte culture**: Conditionally immortalized human podocytes (PDs) were generated by introducing temperature-sensitive simian virus 40 T antigen [29]. At a temperature of 33 °C, these proliferate; at a temperature of 37 °C, they enter the growth arrest phase and become mature podocytes. The differentiation of PD was achieved after 10 days of culture in collagen-coated flask at 37 °C in growth medium containing RPMI 1640 supplemented with 10% fetal bovine serum (FBS), 1× penicillin-streptomycin, and 1 mM l-glutamine.

**Human embryonic kidney (HEK) cells**: HEK cells were purchased from ATCC (Manassas, VA, USA) (catalog number CRL-1573TM) and cultured in Dulbecco’s modified Eagle’s medium supplemented with 10% fetal calf serum and 25 mmol/L HEPES.

**miR193a transgenic mice:** miR193a transgenic mice (on a BALB/C background) were kindly gifted by Prof. Christoph A Gebeshuber, Medical University of Vienna, Vienna, Austria. Four-week-old male wild-type BalbC or miR193a transgenic mice (BALB/C background; *n* = 6) were fed Doxycycline in their water (1.5 mg/mL) for four weeks. At the end of the experimental period, blood samples were collected, and kidneys were harvested. Proteins were extracted as described previously [16] and probed for different proteins, including renin, VDR, and podocyte molecular makers. Stored blood samples were assayed for Ang II levels.

**Induction of APOL1 G0, G1, and G2**: The retroviral-induced expression technique was used to generate stable podocyte cell lines for APOL1G0, APOL1G1, and APOL1G2 [30]. These cells were seeded on collagen-coated plates and differentiated by incubation in normal RPMI for 10 days at 37 °C. We used differentiated podocytes (DPDs), including vector (V/DPD)-, APOL1G0 (G0/DPD)-, APOL1G1 (G1/DPD)-, and APOL1G2 (G2/DPD)-podocytes, in the majority of experiments, unless specified otherwise.

**Western Blotting**: Changes in protein expression were studied using Western blot, as described previously [27]. Briefly, protein lysates were prepared in RIPA buffer containing 50 mM Tris-Cl (pH 7.5), 150 mM NaCl, 1 mM EDTA, 1% NP-40, 0.25% deoxycholate, 0.1% SDS, 1 × protease inhibitor cocktail (Cocktail Set I; Calbiochem, San Diego, CA, USA), 1 mM PMSF, and 0.2 mM sodium orthovanadate. After quantifying the protein content using the Bio-Rad Protein Assay kit (Pierce, Rockford, IL, USA), approximately 30 μg/lane was loaded onto 10–12% polyacrylamide (PAGE) precast gels (Bio-Rad, Hercules, CA, USA). Resolved protein bands were transferred to PVDF membranes and incubated with primary antibodies against APOL1 (#66124-I-IG, Proteintech, Rosemont, IL, USA), renin (#SC-133145, Santa Cruz Biotechnology, Dallas, TX, USA), VDR (#Ab3508, Abcam, Cambridge, MA, USA), CD2AP (#SC-25272; Santa Cruz Biotechnology), Nephrin (#Ab235903, Abcam), WT1 (#Ab15249, Abcam), Podocalyxin (#PA-1-46170, Invitrogen, Waltham, MA, USA), BASP1 (#SC-66994, Santa Cruz Biotechnology), HDAC1 (#05-100-I, Millipore, Sigma, Sigma Aldrich, St Louis, MO, USA), EZH2 (#07-1562, Millipore Sigma), and H3K27me^3^ (#9773P, Cell Signaling Technology, Danvers, MA, USA), followed by incubation of horseradish peroxidase-labeled appropriate secondary antibodies. Expression levels of actin/GAPDH proteins were probed as housekeeping genes loading controls using a monoclonal β-actin/GAPDH antibody (#SC-47724, Santa Cruz Biotechnology) on the same (stripped) Western blots. The blots were developed using a chemiluminescence detection kit (Pierce) and scanned on the Bio-Rad ChemiDoc MP imaging system using Image Laboratory software 3.2.1. A change in protein expression was defined as relative fold change normalized for GAPDH/actin.

### 2.1. Renal Histology and Immunofluorescence Studies

Renal cortical sections of control (BALB/C) and miR193aTr mice (*n* = 6) were stained with periodic acid-Schiff (PAS), as described previously [16]. Ten glomeruli in each mouse were examined for glomerular sclerotic lesions.

Renal cortical sections of the mice noted above were labeled for VDR and renin, as described in our previous publication [16]. In brief, cells were co-labeled with VDR (dilution 1:100; anti-rabbit, #Ab3508, Abcam) and renin (dilution 1:250; anti-mouse, #SC-133145, Santa Cruz Biotechnology). DAPI was used for nuclear localization. The cortical sections of control and miR193aTr mice were examined under a confocal microscope.

### 2.2. Immunoprecipitation Studies

Lysates from V/DPDs, G0/DPDs, G1/DPDs, and G2/DPDs were immunoprecipitated after adding 5 μg of WT1 antibody (Abcam). The immune complexes were collected using 25 μL of protein −A + G sepharose beads (GE Health Care, Life Sciences, Marlborough, MA, USA) in radioimmunoprecipitation assay (RIPA) buffer. IP was carried out at 4 °C for 4 h on a rotating platform. Following this, precipitated A/G proteins were pelleted down by centrifugation at 4500 rpm for 10 min at 4 °C. Next, the protein pellet was washed (3×) each time with 1 mL of cold RIPA lysis buffer, followed by centrifugation each time for 10 min at 2500 rpm in a microfuge. After washings, beads were re-suspended in 100 μL of lysis buffer, to which (sodium dodecyl sulfate-polyacrylamide gel electrophoresis (SDS-PAGE) sample buffer (50 μL) was added, and samples were heated at 100 °C. This was followed by SDS-PAGE, and they were immunoblotted using specific antibodies as indicated.

### 2.3. ANG II ELISA

ANG II levels were determined in the stored plasma of control (BALB/C and FVB/N) and experimental mice (miR193aTr and APOL1G0, APOLG1, and APLOLG1/2 (details of these mice have been described in our previous publication, ref. [20])) using commercial ELISA kits (Peninsula Laboratories, Belmont, CA, USA) as described by the manufacturer. Briefly, ANG II was extracted with 20 mM Tris buffer, pH 7.4, and partially purified and concentrated after filtering through Centricon Filters (MW cut-off 10,000, Millipore, Billerica, MA, USA).

### 2.4. RNA Isolation and qRTPCR Studies

Total cellular RNA was isolated using TRIzol reagent (Invitrogen). Step real-time PCR (iTaq Universal SYBR Green kit; Bio-Rad Laboratories) was performed to analyze the change in expression using a 20 µL reaction mix (containing iTaq Universal SYBR Green reaction mix, script reverse transcriptase, forward and reverse primers, RNA, and nuclease-free water). qPCR was performed at 50 °C for 10 min and at 95 °C for 1 min, followed by 40 cycles of 95 °C for 15 s and at 60 °C for 1 min in the Roche 480 Light Cycle system. Relative gene expression was calculated using the ΔΔCT method and expressed as fold change normalized to the endogenous reference gene, GAPDH.

### 2.5. MicroRNA Assay

The miR193a and U6-small nuclear RNA (U6snuRNA)-specific RT primers were used to generate first-strand cDNA with the help of the TaqMan microRNA Reverse Transcription kit (ThermoFisher Scientific), following the manufacturer’s instruction. An miRNA PCR reaction (15 ul) was prepared containing 100 mM dNTP mix (0.15 µL), multiscribe RT enzyme 50 U/µL (1 µL), 10 × RT buffer (1.5 µL), RNase inhibitor 20 U/µL (0.19 µL), nuclease-free water (4.16 µL), RNA (5 µL), and primers (3 µL). Synthesis was performed under the following conditions: 16 °C for 30 min, at 42 °C, at 85 °C for 5 min, and at 4 °C until stopped in an ABI 7500 (Applied Biosystems, ThermoFisher, Waltham, MA, USA). For qRTPCR, we mixed TaqMan PCR master mix II (5 µL), cDNA (2 µL), nuclease-free water (2 µL), and primer (1 µL) and amplified it at 50 °C for 2 min at 95 °C for 10 min, followed by 40 cycles of 95 °C for 15 s at 60 °C for 1 min. U6 was used as an internal control. Relative quantification of gene expression was calculated using the ΔΔCT method, and the results were normalized to U6-snuRNA expression.

### 2.6. Transfection of APOL1 and miR193a Expression Plasmid

APOL1 and miR193a-based transfections were conducted using published protocols [16,19]. miR193a expression plasmid (25 nM; #SC400232; OriGene, Rockville, MD, USA), APOL1 plasmid (25, 50 and 100 ng), or an empty vector (25 nM; pCMV-MIR; OriGene, Rockville, MD USA) was transfected into the cells using Lipofectamine 3000 Transfection Reagent (ThermoFisher Scientific, Waltham, MA, USA). Briefly, 70–80% confluent differentiated, Rockville, MD, USA podocytes (DPDs) were transfected using Lipofectamine transfection reagent (7.5 µL) and plasmid DNA diluted in Opti-MEM (125 and 250 µL; Applied Biosystems, ThermoFisher Scientific), followed by the addition of P3000 Enhancer Reagent (10 µL) to the diluted DNA This mixture was incubated for 10 min at room temperature (25 °C) and added to the cell. Control and transfected cells were harvested for protein and RNA analyses.

### 2.7. VDR Overexpression with the Treatment of Vitamin D Receptor Agonist

VDR expression was induced using a Vitamin D receptor agonist (VDA; EB 1089, 10 nM; Tocris Bioscience, Bristol, UK). The VDA mixture was prepared in 10% DMSO (100 µL) and used at a final concentration of 10 to 1 uM after dilution in PBS buffer (pH 7.2). Cells were treated for 48 h with VDA or vehicle (DMSO) and analyzed for the change in protein expression level. The DMSO concentration was kept at 0.1% in the vehicle experiments.

### 2.8. Silencing of BASP1

Podocytes (PDs) were transfected with scrambled siRNA (control) and siRNA-BASP1 (20 nM; Santa Cruz Biotechnology) with Lipofectamine RNAiMAX transfection reagent according to the manufacturer’s protocol (ThermoFisher). Briefly, PDs were transfected at 60–80% confluence into a six-well plate. Lipofectamine reagent (9 µL) and siRNAs (10 µM, 2–3 µL) were diluted in opti-MEM media (150 µL) (ThermoFisher). Then, diluted siRNA (150 µL) was added to diluted Lipofectamine reagent (150 µL) in a 1:1 ratio (*v*/*v*) and incubated for 5 min at room temperature (25 °C). After incubation, the siRNA lipid complex was added to cells and kept at 37 °C in opti-MEM media for 48 h. The cells were harvested for protein and RNA analyses.

**Luciferase assay:** A LUC-pair duo-luciferase assay was conducted to validate the binding of miR193a-5p to VDR 3′ UT. We used VDR wild-type 3′ UTR (pEZX-MT06-VDR, HmiT018474) and pEZX-MT06-Control (scrambled non-specific sequence 3′UTR control vectors, CmiT000001-MT06) containing firefly luciferase and Renilla luciferase as tracking genes, which were purchased from GeneCopoeia Inc. (Rockville, MD, USA). The Luc-Pair™ Duo-Luciferase HS Assay Kit measured the relative luciferase activity described in the manufacturer’s protocol. Briefly, cells were transiently co-transfected by using Lipofectamine 2000 with wild-type or control reporter 3′-UTR plasmids and miR-193a (pCMV-miR-193a) or negative miR (control, AM17110) in the combination described. After 48 h of co-transfection, the firefly luciferase activities were measured using the Duo-Luciferase HS Assay (GeneCopoeia). The relative luciferase activity was calculated by normalizing it to Renilla luciferase.

### 2.9. Renin Promoter Identification

We used the Eukaryotic Promoter Database (EPD) [31] to search for the renin promoter sequence, and then we used 3DNA [32] for the protein interaction site; there could be multiple protein binding sites on the renin promoter sequence.

### 2.10. Homology Modeling and Docking

The ITasser tool [33] was used for a template-based approach to generate homology models of WT1, BASP1, EZH2, and HDAC1. The 3-dimensional structure model of the renin promoter was generated using 3DNA [32], which uses all possible canonical and non-canonical base pair geometries and is based on sequence-specific base pair step and base pair rigid body parameters and template coordinates to generate a 3-dimensional structure model. The Galaxy Refine tool refined all the protein models [34]. The refinement process involved repetitive relaxations by short molecular dynamics simulations for mild (0.6 ps) and more robust (0.8 ps) relaxations with 4 fs time steps after structure perturbation. Some specific structural parameters were improved by refinement, i.e., increased residues were favored in Ramachandran plots, and poor rotamere was decreased. The DNA-protein complexes were generated using the docking approach. The HDOCK tool [35], which uses a shape-based pairwise scoring function, was used to create the renin promoter and WT1-BASP1 complex, and further, the Patchdock tool [36], which uses a geometry-based molecular docking algorithm and calculates docking transformations with molecular shape complementarity and atomic desolvation energies, was used to generate the repressor complex of WT1-BASP1-EZH2-HDAC1 bound to the renin promoter.

### 2.11. Protein-Protein Interactions

The protein-protein interactions of the WT1-BASP1-EZH2-HDAC1 complex were calculated using PDBePISA [37].

### 2.12. Visualization

All the protein and DNA structure models and protein-protein complexes were visualized using PyMOL, as well [38].

### 2.13. Statistical Analysis

Statistical comparisons were performed with the program PRISM using unpaired *t*-tests. Two-way ANOVA was used to compare multiple columns. *p* < 0.05 was accepted as statistically significant.

## 3. Results

### 3.1. Effect of APOL1 Risk and Non-Risk Alleles on Podocyte Renin, VDR, Podocyte Molecular Markers (PDMMs), and miR193a Expression

**Figure 1:**
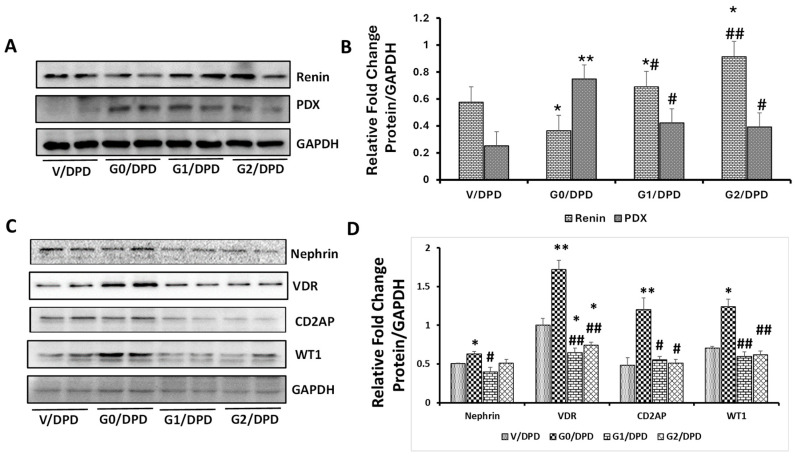
Effect of APOL1 risk and non-risk alleles on podocyte expression of VDR, renin, and PDMMs. (**A**) Podocytes expressing Vector, G0, G1, and G2 were differentiated for 10 days. Differentiated podocytes (DPD) were harvested, and proteins were extracted and probed for renin and Podocalyxin (PDX) and reprobed for GAPDH (*n* = 4). Representative gels from two different lysates are shown. (**B**) Cumulative densitometric data (*n* = 4) are shown in bar graphs. Renin: * *p* < 0.05 vs. V/DPD; # *p* < 0.05 vs. G2/DPD; ## *p* < 0.01 vs. G0/DPD.PDX: ** *p* < 0.01 vs. VD/DPD; # *p* < 0.05 vs. G0/DPD. (**C**) The lysates mentioned above (**A**) were also probed for Nephrin, VDR, CD2AP, WT1, and GAPDH (*n* = 4). Representative gels from two different lysates are displayed. (**D**) Cumulative densitometric data (*n* = 4) are shown in a bar diagram. Nephrin: * *p* < 0.05 vs. V/DPD, G1/DPD, and G2/DPD; # *p* < 0.05 vs. V/DPD. VDR: ** *p* < 0.01 vs. V/DPD, G1/DPD, and G2/DPD; * *p* < 0.05 vs. V/DPD; # *p* < 0.05 vs. V/DPD; ## *p* < 0.01 vs. G0/DPD. CD2AP: ** *p* < 0.01 vs. V/DPD; # *p* < 0.05 vs. G0/DPD. WT1: * *p* < 0.05 vs. V/DPD; ## < 0.01 vs. G0/DPD.

Cellular lysates from differentiated podocytes (DPDs) expressing vector (V/DPD), APOL1G0 (G0/DPD), APOL1G1 (G1/DPD), and APOLG2 (G2/DPDs) were probed for renin, Podocalyxin (PDX), and GAPDH. Representative gels are displayed in Figure 1A. Cumulative densitometric data are shown in Figure 1B. A significantly lower level (*p* < 0.05) of renin was observed in podocytes expressing G0/DPDs when compared with V/DPDs; in contrast, G1/DPDs (*p* < 0.05) and G2/DPDs (*p* < 0.01) exhibited a higher expression of renin when compared to V/DPDs. However, the expression of PDX was significantly increased in G0/DPDs when compared to V/DPDs (*p* < 0.01), G1/DPDs (*p* < 0.05), and G2/DPDs (*p* < 0.05).

Cellular lysates were further probed for VDR, Nephrin, CD2AP, WT1, and GAPDH. Representative gels are displayed in Figure 1C. Cumulative densitometric data are shown in a bar diagram (Figure 1D). Nephrin was significantly elevated in G0/DPDs when compared to V/DPDs (*p* < 0.05), G1/DPDs (*p* < 0.05), and G2/DPDs (*p* < 0.05). Similarly, G0/DPDs displayed elevated VDR expression when compared to V/DPDs (*p* < 0.01), G1/DPDs (*p* < 0.01), and G2/DPDs (*p* < 0.01). G0/DPDs also exhibited a higher expression of CD2AP expression in comparison to V/DPDs (*p* < 0.05), G1/DPDs (*p* < 0.05), and G2/DPDs (*p* < 0.05). As expected, the expression of the master transcriptor-WT1- was also elevated in G0/DPDs when compared to V/DPDs (*p* < 0.05), G1/DPDs (*p* < 0.05), and G2/DPDs (*p* < 0.05).

**Figure 2:**
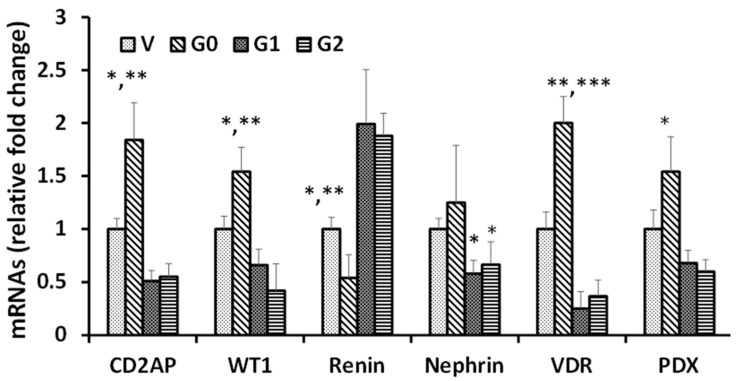
Podocyte mRNA level alterations in APOL1 milieus. To determine mRNA levels of APOL1-expressing podocytes, RNAs were extracted from cellular lysates V/DPDs, G0/DPDs, G1/DPDs, and G2/DPDs (*n* = 4). cDNAs were amplified using specific primers (CD2AP, WT1, renin, VDR, and PDX [podocalyxin]). CD2AP: * *p* < 0.05 with respective V and ** *p* < 0.01 with respective G1 and G2; WT1: * *p* < 0.05 with respective V and ** *p* < 0.01 with respective G1 and G2; Renin: * *p* < 0.05 with respective G0 and ** *p* < 0.01 with respective G1 and G2; Nephrin: * *p* < 0.05 with respective V and G0; VDR: ** *p* < 0.01 with respective V and *** *p* < 0.001 with respective G1 and G2; PDX: * *p* < 0.05 with respective V, G1, and G2.

To analyze whether the alteration in protein expression was mediated through modulation of mRNA levels of these proteins in APOL1 milieus, RNAs were extracted from cellular lysates of V/DPDs, G0/DPDs, G1/DPDs, and G2/DPDs (*n* = 4). cDNAs were amplified using specific primers of podocyte molecules. The primer sequences used to amplify mRNAs are shown in Table 1. G0 increased the levels of CD2AP, WT1, VDR, and PDX mRNAs when compared to V; in contrast, both G1 and G2 attenuated the expression of CD2AP, WT1, Nephrin, VDR, and PDX mRNAs when compared to both V and G0 (Figure 2).

**Figure 3:**
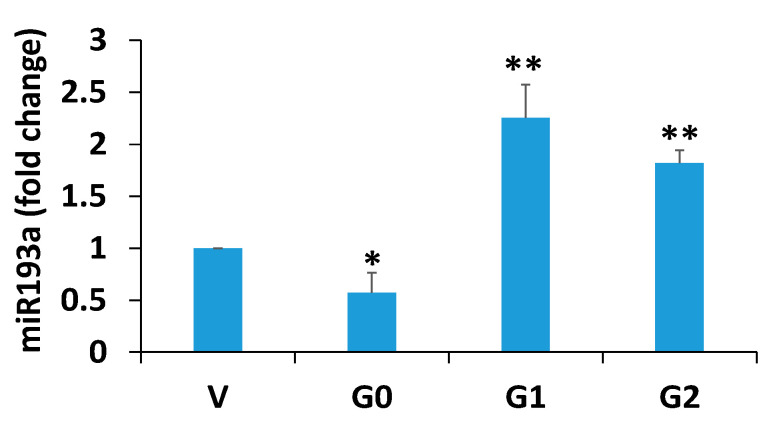
miR1 93a expression in podocytes expressing Vector (V), APOL1G0, APOL1G1, and APOL1G2. Extracted RNAs (*n* = 4) were assayed for miR193a. Results (means ± SD) are displayed in a bar diagram. * *p* < 0.05 vs. V; ** *p* < 0.01 vs. V and G0.

To determine the miR193a levels, RNAs were extracted from V/DPD, G0/DPD, G1/DPD, and G2/DPD and assayed for miR193. G0/DPDs showed down-regulation, but G1/DPD and G2/DPDs displayed enhanced expression of miR193a (V/DPD 1.0, G0/DPD 0.45, G1/DPD 2.5, and G2/DPD 2.8 [miR193a, fold change3]). These findings were consistent with our previously reported results showing disruption of the miR193a axis in podocytes expressing APOL1 risk alleles [16], Figure 3.

### 3.2. Renin-Angiotensin System in APOL1 Milieus

**Figure 4:**
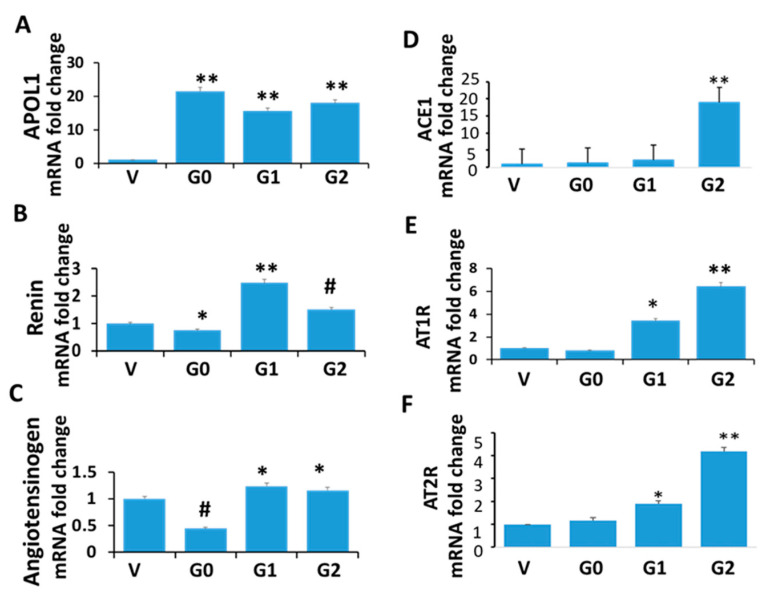
Renin-angiotensin status in APOL1 milieus. RNAs were extracted from V/DPD, G0/DPD, G1/DPD, and G2/DPD (*n* = 4), and cDNAs were amplified with specific primers for APOL1, renin, angiotensinogen, ACE1, AT1R, and AT2R. (**A**) APOL1: ** *p* < 0.01 compared to V. (**B**) Renin: * *p* < 0.05 compared to V; ** *p* < 0.01 compared to V and G0; # *p* < 0.05 compared to V and G1. (**C**) # *p* < 0.05 compared to V, G1, and G2; * *p* < 0.05 compared to V. (**D**) ** *p* < 0.01 compared to V, G0, and G1. (**E**) AT1R: * *p* < 0.05 compared to V, G0, and G2; ** *p* < 0.01 compared to V and G0. (**F**) AT2R: * *p* < 0.05 compared to V and G0; ** *p* < 0.01 compared to V, G0, and G1.

Renin, an enzyme released by the juxtaglomerular apparatus of afferent arterioles, cleaves angiotensinogen (Agt, predominantly generated by the liver but also locally generated by renal cells, including podocytes) to angiotensin I; the latter is cleaved by angiotensin-converting enzyme (ACE) 1 to angiotensin II, which activates angiotensin II Type 1 or 2 receptors (AT1R/AT2R) to initiate downstream signaling [6,7]. To determine the status of the RAS in the APOL1 milieus, RNAs were extracted from cellular lysates of V/DPDs, G/DPDs, G1/DPDs, and G2/DPDs. cDNAs were amplified using specific primers. Cumulative data are displayed in Figure 4. G1 and G2 enhanced angiotensinogen (Agt), renin, AT1R, and AT2R mRNA expressions when compared to V (Figure 4A–F); in contrast, G0 displayed decreased renin and angiotensinogen mRNA expression when compared to V (Figure 4B,C). G1 and G2 also showed enhanced expression of AT1R and AT2R expression when compared to V and G0 (Figure 4E,F).

### 3.3. WT1-BASP1 Repressor Complex Modulates Renin Expression

**Figure 5:**
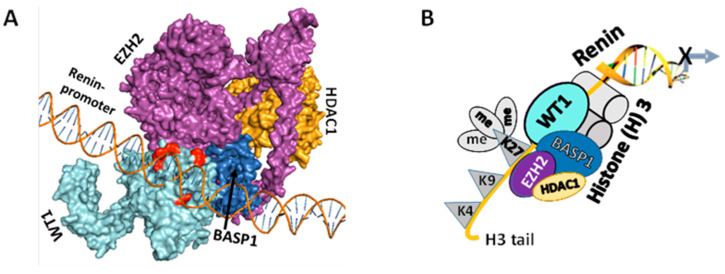
The structural construct of the WT1-BASP1 repressor complex. (**A**) Homology modeling and docking studies suggested the binding of WT1-BASP1 repressor complex on the renin promoter. (**B**) A schematic diagram displays the formation of the WT1-BASP1 repressor complex at the renin promoter. WT1 recruits BASP1, EZH2, and HDAC1, inducing methylation at Lysine 27 residues at Histone (H) 3 tail.

Homology modeling and docking studies suggested the binding of the WT1-BASP1 repressor complex to the renin promoter, as shown in Figure 5A. We proposed that the WT1-BASP1 complex binds with the renin promoter and recruits the methyltransferase EZH2 along with HDAC1, resulting in methylation of Lysine (K) 27 at Histones (H) 3 tail, as shown in the schematic diagram (Figure 5B).

**Figure 6:**
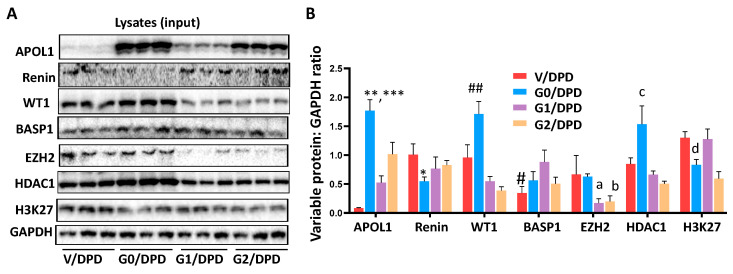
Analysis of input lysates of podocytes expressing APOL1 non-risk and risk alleles. (**A**) Proteins were extracted from the cellular lysates of V/DPDs, G0/DPs, G1/DPDs, and G2/CDPs (*n* = 3–4). Gels from three independent lysates are displayed. (**B**) Cumulative densitometric data of proteins displayed in 3A are shown in a bar diagram. APOL1: ** *p* < 0.01 vs. G1/DPD and G2/DPD; *** *p* < 0.001 vs. V/DPD; Renin: * *p* < 0.05 vs. respective variables; WT1: ## *p* < 0.01 vs. respective variables; BASP1: # *p* < 0.05 vs. respective variables; EZH2: ^a^ *p* < 0.01 vs. V/DPD and G0/DPD; ^b^ *p* < 0.01 vs. V/DPD and G0/DPD; HDAC1: ^c^ *p* < 0.01 vs. respective other variables; H3K27me^3^: ^d^ *p* < 0.01 vs. V/DPD, G1/DPD, and G2/DPD.

To analyze the components of WT1-BASP1 repressor complexes, protein blots of three different cellular lysates (input) of V/DPDs, G0/DPDs, G1/DPDs, and G2/DPDs were probed for WT1, BASP1, EZH2, HDAC1, H3K27me^3^, and GAPDH. The gels of three independent lysates are displayed in Figure 6A. Cumulative densitometric data are shown in bar graphs (Figure 6B).

**Figure 7:**
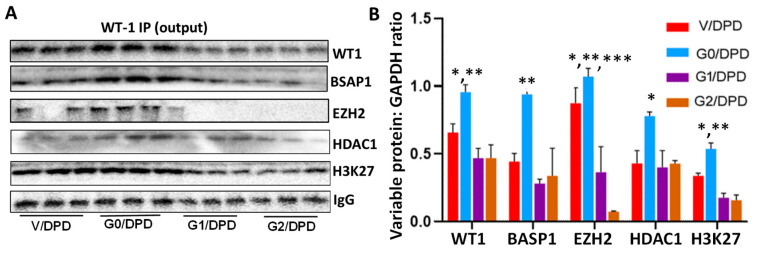
Analysis of WT1 antibody-bound proteins (output). (**A**). Cellular lysates from the protocol of Figure 6A were immunoprecipitated (IP) with the WT1 antibody. Protein blots of WT1-IP fractions were probed for WT1, renin, EZH2, HDAC1, H3K27me^3^, and IgG (*n* = 3). Gels from 3 independent cellular lysates are displayed. (**B**). Cumulative densitometric data from blots of the Figure 7A (*n* = 3). WT1: * *p* < 0.05 vs. V; ** *p* < 0.01 vs. G1 and G2. BASP1: ** *p* < 0.01 vs. respective other variables. EZH2: * *p* < 0.05 vs. V; ** *p* < 0.01 vs. G1; *** *p* < 0.001 vs. G2. HDAC1: * *p* < 0.05 vs. other variables. H3K27: * *p* < 0.05 vs. V; ** *p* < 0.01 vs. G1 and G2.

To validate the components of WT1-BASP1 repressor complexes, the lysates mentioned above were immunoprecipitated with the WT1 antibodies (*n* = 3). Protein blots of WT1-IP fractions (output) were probed for WT1, BASP1, EZH2, HDAC1, H3K27me^3^, and IgG. Gels are displayed in Figure 7A. Cumulative densitometric data are shown in bar graphs (Figure 7B). IP fractions showed enhanced expressions of WT1, BASP1, EZH2, HDAC1, and H3K27me^3^ in G0/DPDs.

**Figure 8:**
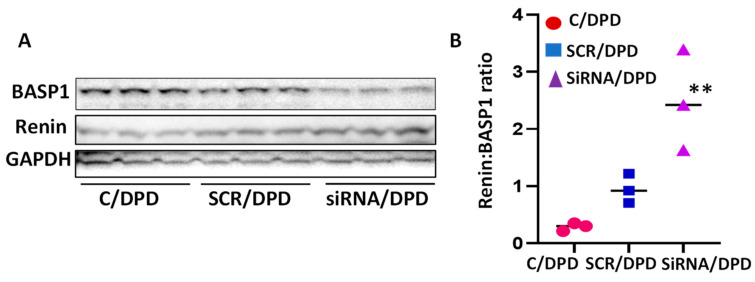
Effect of BASP1 silencing on the podocyte expression of renin. (**A**) Cellular lysates of control podocytes (C/DPD), scrambled siRNA- (SCR/DPD), and BASP1-SiRNA-transfected podocytes (SiRNA/DPD) were probed for renin and GAPDH (*n* = 3). Gels of three independent lysates are shown. (**B**) Cumulative densitometric data (*n* = 3) are shown in bar graphs. ** *p* < 0.01 vs. C/DPD and SCR/DPD.

To confirm the role of BASP1 in WT1 repressor complexes in the suppression of renin in podocytes, DPDs were transfected with either scrambled (SCR) or SiRNA-BASP1. Proteins of cellular lysates of control (C)/DPD, SCR/DPD, and SiRNA/DPD were probed for BASP1, renin, and GAPDH. Three independent gels are displayed in Figure 8A. Cumulative densitometric data are shown in a bar diagram (Figure 8B). BASP1-silenced DPDs showed a higher expression of renin.

### 3.4. Podocytes Overexpressing VDR Showed a Reduction in Renin Expression but Enhanced Expression of Podocyte Molecular Markers (PDMMs)

**Figure 9:**
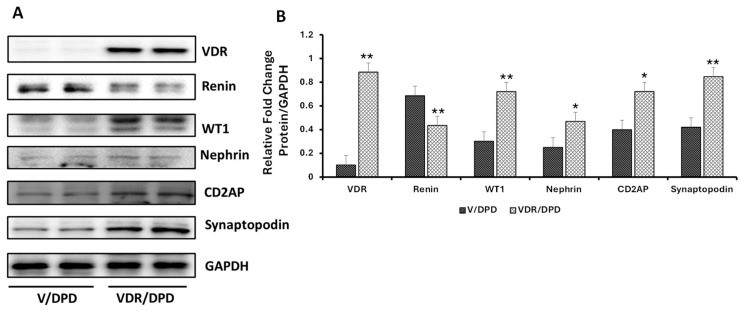
Effect of VDR overexpression on renin and podocyte molecular markers (PDMMs). (**A**) DPDs were incubated in media containing either vehicle (DMSO) or VDA (EB 1089, 10 nM) for 48 h (*n* = 4). Proteins were extracted and probed for VDR, renin, and PDMMs (WT1, Nephrin, CD2AP, and Synaptopodin) and GAPDH. Gels from two different lysates from V/DPD (vehicle-treated) and VDR/DPD (VDA-treated) are shown. (**B**) Cumulative densitometric data for different variables are shown in bar graphs. * *p* < 0.0.5 and ** *p* < 0.01 vs. respective V/DPD.

Vitamin D, as well as VDR agonist, have been demonstrated to attenuate the expression of renin in podocytes [26,27]. We asked whether overexpression of VDR in podocytes would downregulate the expression of renin in podocytes; if so, the reduction in renin would be associated with enhanced expression of the podocyte molecular markers. To generate overexpression of VDR, podocytes were treated with either vehicle (V) or a VDR agonist, and the cellular lysates were probed for VDR and renin and reprobed for molecular markers. Representative gels are displayed in Figure 9A. Cumulative densitometer data are provided in a bar diagram (Figure 9B). Podocytes overexpressing VDR (VDR/DPDs) showed an enhanced expression (*p* < 0.01) of VDR, but decreased expression (*p* < 0.01) of renin when compared to V/DPD. Moreover, VDR/DPDs exhibited increased expression of podocyte molecular markers in comparison to V/DPDs.

### 3.5. Dose-Response Effect of APOL1G0 on Renin Expression

**Figure 10:**
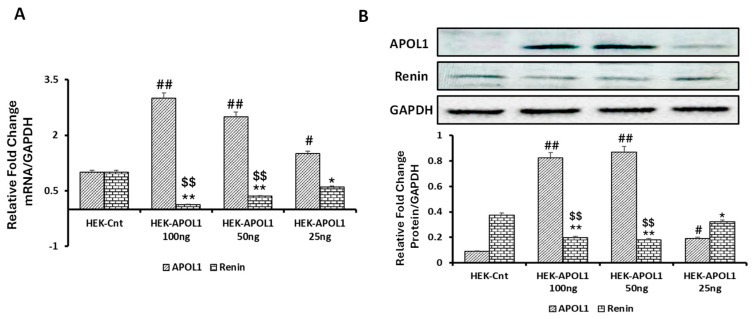
Dose-response effect of APOL1 induction on renin expression in HEK cells. HEK cells were transfected with either empty vector (control, HEK-Cnt) or APOL1 plasmid (HEK-APOL1) in different concentrations (25, 50, and 100 ng) for 48 h (*n* = 4). Cells were harvested, and proteins and RNAs were extracted. (**A**) cDNAs were amplified with specific primers of APOL1 and renin. Cumulative data are shown in a bar diagram. APOL1 expression: # *p* < 0.05 and ## *p* < 0.01 vs. HEK-Cnt. Renin: * *p* < 0.05 and ** *p* < 0.01 vs. HEK control; $$ *p* < 0.01 vs. HEK-APOL1, 25 ng. (**B**) Proteins were probed for APOL1, renin, and GAPDH. Representative gels are displayed in the upper panel. Cumulative densitometric data are shown in bar graphs. APOL1: # *p* < 0.05 and ## *p* < 0.01 vs. HEK-Cnt. Renin: * *p* < 0.05 and ** *p* < 0.01 vs. HEK-Cnt; $$ *p* < 0.01 vs. HEK-APOL1, 25 ng.

We have previously demonstrated that APOLG0 inversely regulates the expression of miR193a in podocytes [14]. Since miR193a also negatively regulates VDR expression in podocytes [15], we hypothesize that APOL1G0 would also inversely modulate the expression of renin in kidney cells. To examine this hypothesis, we transfected different doses of APOL1G0 plasmid (25, 50, and 100 ng) in HEK cells (which do not express APOL1 protein). After 48 h of transfection, the cells were harvested for RNA and protein extraction. Proteins were probed for APOL1, renin, and GAPDH. cDNAs were amplified with specific primers for APOL1 and renin. Data on the relative mRNA profiles of APOL1 and renin are shown in Figure 10A. APOL1-transfected HEKs showed an increase in the mRNA expression of APOL1 in a dose-dependent manner. APOL1 also negatively increased renin mRNA expression in HEKs dose-dependently (Figure 10A).

Representative gels displaying protein expression are shown in the upper panel of Figure 10B. Cumulative densitometric data are shown in the lower panel of Figure 10B. APOL1-overexpressing HEK cells exhibited decreased expression of renin (Figure 10B).

### 3.6. Relationship Between miR193a and VDR

**Figure 11:**
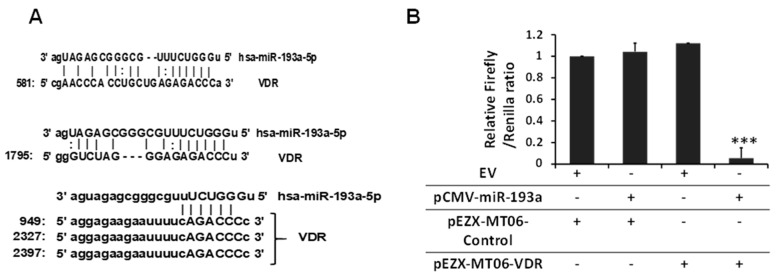
Validation of miR193a putative binding sites on VDR. (**A**) Available online in silico analysis tools (microrna.org; mirdb.org and TargetScan). VDR was predicted as a potential target for miR193a-5p. Predicted binding sites are shown. (**B**) Podocytes were transiently co-transfected by using Lipofectamine 2000 with wild-type or control reporter 3′-UTR plasmids and miR-193a (pCMV-miR-193a) or negative miR (control, AM17110) in combination. After 48 h of co-transfection, the firefly luciferase activities were measured using the duo-luciferase HS assay. The relative luciferase activity was calculated by normalizing it to Renilla luciferase. The presented results are cumulative values of three independent experiments, each performed in triplicate. *** *p* < 0.001 vs. other variables.

Utilizing available in silico analysis tools (microrna.org; mirdb.org and TargetScan 8), VDR was predicted as a potential target for miR193a-5p. Five binding sites of VDR on miR193 were predicted (microrna.org, mirab.org, and TargetScan 8), as shown in Figure 11A.

A LUC-pair duo-luciferase assay was performed to validate the binding of miR193a-5p to VDR 3′ UTR. The presented results are cumulative values of three independent experiments, each conducted in triplicate (Figure 11B). The luciferase assay validated miR193a binding sites on VDR.

### 3.7. Effect of miR193a on the Expression of Podocyte and VDR, Renin, and Podocyte Molecular Markers

**Figure 12:**
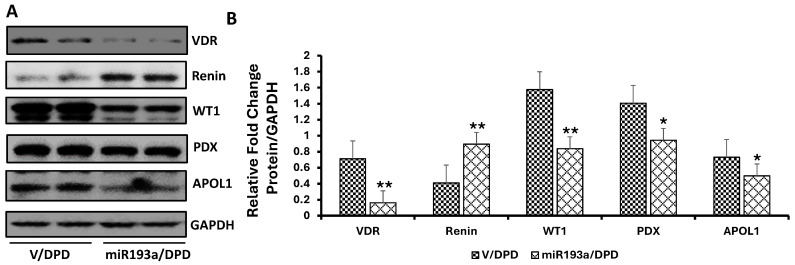
Effect of miR193a on the expression of VDR, renin, and PDMMs. (**A**) Podocytes were transfected with empty vector or miR19a plasmid and differentiated (*n* = 4). Cellular lysates were probed for VDR, renin, PDMMs (WT1, PDX, APOL1), and GAPDH. Gels from two different lysates are shown. (**B**) Cumulative data are shown in bar graphs. * *p* < 0.05 and ** *p* < 0.0.01 vs. respective V/DPD.

We have previously demonstrated that enhanced miR193a expression in podocytes was associated with attenuated VDR mRNA expression, and inhibition of miR193a enhanced VDR mRNA expression in podocytes [15]. Now, we have validated miR193a binding at 3′ UTR of the VDR gene. Because VDR negatively modulates renin expression [26,27], its reduction will activate the RAS and compromise podocyte health. To confirm this notion, podocytes overexpressing miR193a were probed for renin and podocyte molecular markers. Three sets of DPDs were transfected with miR193a; proteins were extracted and probed for VDR and reprobed for Renin, WT1, PDX, and APOL1. Blots were reprobed for GAPDH. Representative gels are displayed in Figure 12A. Cumulative densitometric data are shown in Figure 12B. miR193a/DPDs showed a significant decrease (*p* < 0.01) in VDR and an increase in renin expression when compared to V/DPDs (Figure 12B). miR193a/DPS showed a decrease (*p* < 0.05) not only in APOL1, but also in PDX and WT1 expression when compared to V/DPDs (Figure 12A,B).

### 3.8. Effect of miR193a on the Expression of Podocyte and VDR, Renin, and Podocyte Molecular Markers in Renal Tissues of Control and miR193aTr Mice

**Figure 13:**
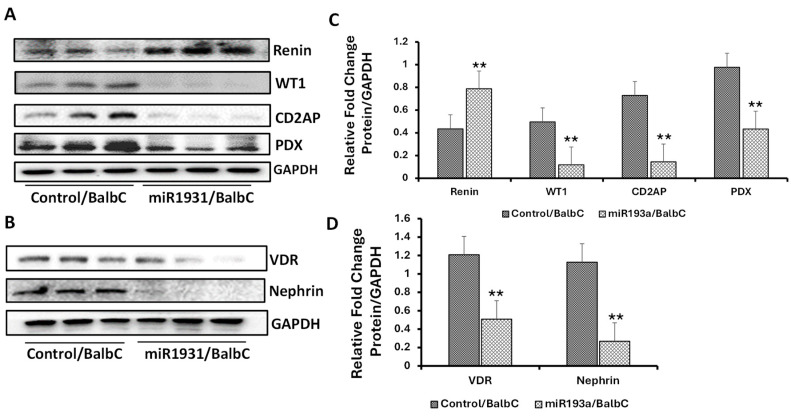
Renal tissue expression profile of VDR, renin, and PDMMs in control and miR193aTr mice. (**A**) Renal tissues were harvested from control (Balb/C, wild-type) and miR193aTr mice (*n* = 3). Proteins were probed for renin, PDMMs (WT1, CD2AP, PDX), and GAPDH. Gels from three different lysates are displayed. (**B**) Tissue lysates from the above mice were reprobed for VDR, Nephrin, and GAPDH (*n* = 3). Gels from different lysates are shown. (**C**) Cumulative densitometric data from gels are shown in panel A in bar graphs. ** *p* < 0.01 vs. control/Balb C mice. (**D**) Cumulative densitometric data from gels displayed in Panel B are shown in a bar diagram. ** *p* < 0.0.01 vs. control/Balb C mice.

Patients with idiopathic FSGS have been shown to display enhanced glomerular expression of miR193a [17]. miR193aTr mice are also an experimental animal model of idiopathic FSGS [17]. Based on our cellular data, we hypothesized that renal tissues of miR193aTr mice would display reduced VDR but increased expression of renin. To validate this hypothesis, proteins were extracted from the renal tissues of control (Balb/C, *n* = 3) and experimental (miR193aTr, *n* = 3) mice and probed for renin, WT1, CD2AP, PDX, and GAPDH. Representative gels from three different lysates are shown in Figure 13A.

Cumulative densitometric data are shown in bar graphs (Figure 13C). The same lysates were also probed for Nephrin, VDR, and GAPDH. Gels from three different lysates are shown in Figure 13B. Cumulative densitometric data are shown in Figure 13D. miR193aTr mice showed enhanced renal tissue expression of renin and an attenuated expression of VDR (Figure 13B,D). The expression of podocyte markers (WT1, CD2AP, PDX, and Nephrin) was also lower in the renal tissues of miR193aTr mice compared to control mice (Figure 13C,D).

### 3.9. Renal Histology and Immunofluorescence Labeling for VDR and Renin in Renal Cortical Sections of Control and miR193aTr Mice

**Figure 14:**
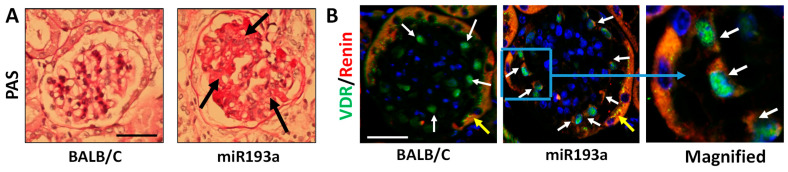
Renal histology and VDR/renin expression in control (BALB/C) and miR193aTr mice. (**A**) Representative glomeruli from a control and miR193aTr mice. Sclerosis is displayed by black arrows in a glomerulus from an miR193aTr mouse. (**B**) Glomeruli were co-labeled with VDR and renin antibodies. Nuclei were stained with DAPI. A representative glomerulus from a control mouse showed co-labeled (VDR and renin) podocytes (white arrows); podocytes predominantly displayed green fluorescence (VDR) and minimal red fluorescence (renin). A representative glomerulus from an miR193aTr mouse displayed both green (VDR) and red (renin) fluorescence in podocytes (white arrows). Parietal epithelial cells showed orange fluorescence (combination of predominant red and mild green fluorescence, indicated by yellow arrows) in glomeruli from both BALB/C and miR193aTr mice. C. Blue square is magnified to display co-labeling of VDR and renin in podocytes. Scale bar = 50 µM.

We carried out PAS staining on renal cortical sections of BALB/C and miR193aTr mice to confirm the development of glomerulosclerosis. A representative glomerulus from a control (BALB/C) and an miR193aTr mouse are displayed in Figure 14A. Capillary loops in the glomeruli from an miR193aTr mouse displayed overt sclerosis. To examine the expression of VDR and renin in the kidney, renal cortical sections were immunolabeled for VDR and renin. Nuclei were stained with DAPI. Representative glomeruli from control and miR193aTr mice are shown in Figure 14B. Podocytes in the glomerulus from a BALB/C mouse predominantly showed VDR expression and hardly any expression of renin; in contrast, podocytes in the glomerulus from an miR193aTr mouse displayed labeling for both VDR and renin (green and red fluorescence).

### 3.10. Ang II Blood Levels in Control and Experimental (miR193aTr and APOL1 G0, APOL1G1, and APOL1 G1/G2) Mice

**Figure 15:**
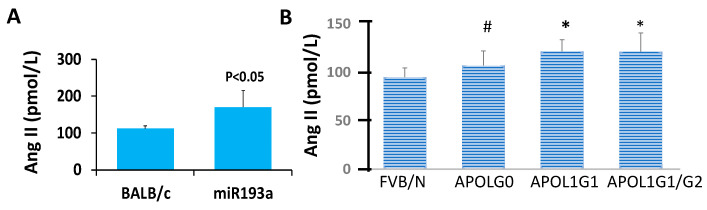
Ang II levels were determined in plasma samples of control (BALB/C, *n* = 6 and FVBN, *n* = 9) and experimental (miR193aTR, *n* = 6; APOL1 G0, *n* = 10; APOL1 G1, *n* = 9; and APOL1G1/G2, *n* = 9) mice. Results (means ± SD) are shown in bar diagrams. (**A**) Plasma Ang II levels in control (BALB/C) and miR193aTr mice. (**B**) Plasma Ang II levels in control and APOL1 mice. * *p* < 0.05 vs. FVBN; # *p* < 0.05 vs. APOL1G1.

Blood Ang II levels were higher (*p* < 0.05) in miR193aTr mice than control mice (BALB/C, 79.99 ± 5.54 vs. miR193aTr, 125.59 ± 38.35 pmol/L), as shown is Figure 15A.

Ang II levels in control (FVB/N), APOL1G0, APOLG1, and APOL1G1/G2 mice are shown in Figure 15B. APOLG1 (121.51 ± 12.08 pmol/L) and APOL1G1/G2 (121.26 ± 19.15 pmol/L) mice showed higher (*p* < 0.05) levels of Ang II when compared to control (FVB/N, 94.83 ± 9.6 pmol/L) mice. There was no difference between the control and APOL1G0 mice (107.03 ± 15.1 pmol/L). APOL1G0 mice showed lower (*p* < 0.05) blood levels of Ang II when compared to APOL1G1 mice.

### 3.11. Role of APOL1 Dynamics in the RAS Activation and Glomerular Sclerosis

**Figure 16:**
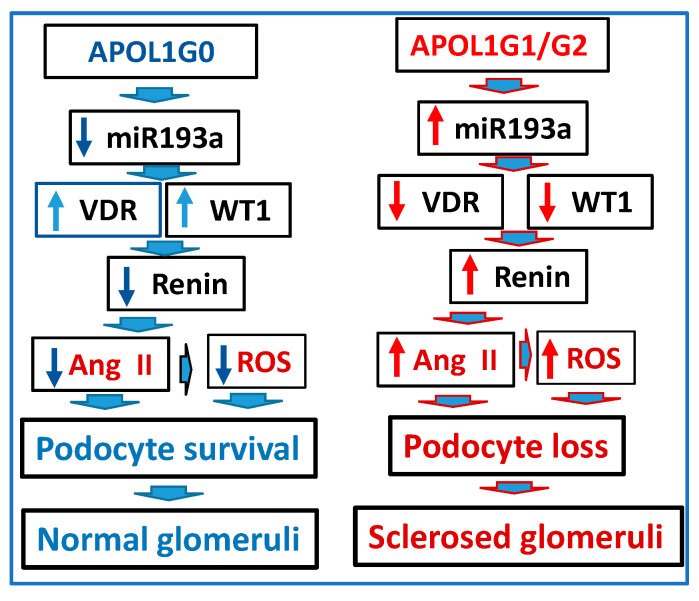
A schematic diagram displaying the activation of the RAS contributing to glomerular sclerosis in APOL1 milieus.

A schematic diagram delineating modulation of the RAS and associated risk of developing FSGS in APOL1 non-risk and risk milieus is shown in Figure 16. Enhanced expression of G0 led to a reduction in miR193a (because of the functional APOL1-miR193a axis, ref. [16]), which enhanced the expression of WT1 and VDR. Both WT1 and VDR led to reductions in renin. They were associated with the attenuation of Ang II generation, which contributed to a decreased release of reactive oxygen species (ROS) and preserved podocyte health. In contrast, the elevated expression of G1 and G2 enhanced the expression of miR193a (because of disruption of APOL1-mIR193a axis, ref. [16]), resulting in attenuation of the expression of WT1 and VDR and associated escalation of renin levels and activation of the RAS, causing escalation of ROS generation in podocytes. Enhanced levels of Ang II and ROS would contribute to the loss of podocytes, resulting in the development of glomerulosclerosis. Interestingly, the VDR deficit will exacerbate, but an optimal level of VDR expression can downregulate miR193a-mediated adverse effects.

## 4. Discussion

The current study demonstrates that podocytes expressing non-risk APOL1 (G0) have lower expression of miR193a and renin, but a higher expression of VDR. In contrast, podocytes expressing APOL1 risk alleles (G1 and G2) display higher expression of miR193a and renin, but a lower expression of VDR when compared to control podocytes.

As expected, podocytes treated with VDA (VDR/DPDs) displayed an enhanced expression of VDR. However, they exhibited a reduction in renin, further confirming the role of VDR in the modulation of renin expression in podocytes. In silico computational algorithms predicted the miR193a target sites in the VDR 3′ UTR region. A putative interaction between miR193a and VDR was validated by the luciferase-VDR reporter assay. Moreover, podocytes overexpressing miR193a showed attenuated VDR expression and enhanced renin expression. These findings also point to the role of miR193a in downregulating VDR with concomitant upregulation of renin. In in vivo studies, renal tissues of miR193aTr mice showed downregulation of VDR and enhanced expression of renin, also confirming the role of miR193a in the downregulation of VDR and upregulation of renin. Homology modeling, docking, and WT-1 immunoprecipitation studies suggest that WT1 forms a repressor complex containing BASP1, EZH2, HDAC1, and H3K27me^3^, which binds at the renin promoter. G0/DPDs displayed an enhanced expression of all the constituents of the WT1-BASP1 complex when compared to G1/DPDs and G2/DPDs. Silencing BASP1 in DPDs correlated with an increase in the expression of renin, further confirming the role of the WT1-BASP1 repressor complex in regulating the VDR-RAS interplay system. Since HEK cells expressing APOLG0 also decreased both protein and mRNA expression of renin, this further confirms the role of APOL1 in renin modulation in kidney cells. Podocytes expressing miR193a and renal tissue extracts from miR193aTr mice showed decreased VDR and increased renin expression associated with attenuated expression of PDMMs. Renal histology confirmed sclerosed glomeruli in cortical sections of miR193aTr mice. Compared to control mice, immunofluorescence studies displayed enhanced renin expression by podocytes in renal cortical sections of miR193aTr mice. Interestingly, miR193aTr mice as well as APOL1G1 and APOL1G1/G2 mice showed higher blood levels of Ang II than control mice. In a nutshell, in an adverse milieu (elevated levels of miR193a), a reduction in VDR was associated with an upregulation of renin and attenuation of podocyte molecular markers; inversely, in a supportive milieu, VDR expression was high (because of the reduced levels of miR193a), and the expression of renin was attenuated and was associated with upregulation of the expression of podocyte molecular markers. The present study also provides evidence of the activation of the RAS in miR193aTr, APOL1G1, and APOL1G1/G2 mice.

In vitro studies demonstrate that WT1 has binding sites at the renin gene in podocytes, and they repress the transcription of renin [20]. These investigators suggested that binding WT1 to the cis-element inhibited basal renin promoter activity, altering renin gene transcription. Renin and WT1 mRNA have been shown in afferent arterioles and glomeruli in mouse kidneys [20]. Also, cultured As4.1 cells (juxta glomerular cell lines) displayed renin and WT1 protein expression. In an experimental hypoxic rat model, podocytes displayed enhanced expression of WT1 (both protein and mRNA) [39]. In the present study, homology modeling and docking studies suggested the binding of the WT1-BASP1 repressor complex on the renin promoter. Analysis of the WT1-IP fraction confirmed the presence of the co-repressor BASP1 and other constituents, including EZH2, HDAC1, and H3K27me^3^. Podocytes expressing G0 showed an enhanced expression of WT1 and increased expression of all the constituents of the WT1-BSAP1 complex when compared to podocytes expressing G1 or G2. Since silencing BSAP1 in podocytes is inversely related to renin expression, this further confirmed the role of BSAP1 as a co-repressor.

WT1 expression has been shown to enhance the expression of VDR four-fold in HEK cells [28]. We have previously demonstrated that treatment of HEK cells and glomerular parietal epithelial cells with a VDR agonist increased the expression of VDR and enhanced the expression of WT1 [19]. In the present study, overt expression of VDR not only increased the expression of WT1, but also downregulated the expression of renin in podocytes. Since both VDR and WT1 function upstream of renin, both might have contributed to the downing of the renin in overtly VDR-expressing podocytes.

In earlier studies, we have shown that VDR heterodimerizes with RXR, forming a VDR-RXR complex, and RXR binds to the miR193a gene [15]. Since silencing of either VDR or RXR enhanced the expression of miR193a in podocytes, it confirmed the role of VDR-RXR heterodimer in the downing of miR193a expression in podocytes. In the present study, we validate the role of miR193a in regulating VDR. These findings suggest that the miR193a-VDR axis plays a bifunctional role in podocytes. However, further studies are needed to confirm the relationship between these molecules.

APOL1 is a membrane channel for transporting monovalent and divalent ions [40,41,42]. These channels are selective for anions at a lower pH, but serve as non-selective cation channels at a neutral pH [41]. The expression of APOL1 risk alleles in human embryonic kidney cells is associated with enhanced K^+^ efflux [40]. We have recently demonstrated that APOL1 risk alleles alter the predicted configuration of cation channel structure in lipid membranes [43]. Overt expression of either APOL1G1 or APOL1G2 in podocytes also increases K^+^ efflux. Interestingly, K^+^ efflux induces hyperpolarization of the cell [44], which enhances renin expression [45]. Therefore, APOL1G1 and G2 may enhance renin expression in podocytes by inducing K^+^ efflux.

Earlier, we reported the existence of a bifunctional APOL1 miR193a axis in kidney cells [19]. Both APOL1 and miR193a inversely regulate each other. However, this relationship is disrupted in podocytes carrying APOL1 risk alleles; on that account, G0/DPDs have a downregulating effect on miR193a, and G1/DPDs and G2/DPDs show an elevation of miR193a levels [16]. The present study confirms, extends, and clarifies this formulation with the involvement of the WT1-BASP1 complex.

Podocyte health is often judged by the optimal expression of podocyte molecular markers [46,47]. We have also evaluated these makers in every experimental protocol in the present study. Enhanced expression of renin by DPDs was always associated with attenuated expression of podocyte molecular markers such as WT1, Nephrin, Podocalyxin (PDX), and CD2AP. Increased renin expression is known to activate the RAS and is associated with podocyte injury, both in vitro and in vivo, in humans and experimental animal studies [48,49,50,51,52]. We have previously reported the role of VDR in activating the renin-angiotensin system in podocytes [53,54]. Vitamin D, which also enhances the transcription of VDR, is a known negative regulator of renin in kidney cells [26,55,56]. Since WT1 is a master transcriptor for podocyte genes such as Nephrin and Podocalyxin [17,18], its downregulation due to direct binding by miR193a also contributes to attenuated podocyte expression of Nephrin and Podocalyxin in APOL1 risk milieu.

Gebeshuber et al. [17] and colleagues published a pioneering paper on the role of miR193a in developing idiopathic FSGS in humans and animal models. The authors attributed the loss of podocytes to the excessive generation of ROS due to escalated levels of miR193a milieu. Interestingly, podocytes express miR19a minimally, and their phenotypes depend on their lack of the expression of miR193a [18]. In contrast, higher levels of miR193a expression are characteristic of parietal epithelial cells [18]. Immunofluorescence studies in the present paper also showed robust expression of renin by parietal epithelial cells in both control and miR193aTr mice, which may be a consequence of lowered VDR in escalated levels of miR193a in parietal epithelial cells. In the present study, miR193aTr mice showed decreased podocyte expression of VDR and overt expression of renin; as expected, they also showed higher levels of Ang II in their blood. Renal histology in miR193aTr mice also confirmed the development of FSGS. Since Ang II has been reported to directly, as well as through the generation of ROS, cause the loss of podocytes [53,54], RAS activation could also have partially contributed to the development of FSGS in the miR193aTr mouse model. In future studies, it would be worthwhile to use inhibition of the RAS to prevent or slow the progression of FSGS in this model. These studies will establish a cause-and-effect relationship between the role of the RAS and the miR193a-mediated development of FSGS.

In an investigative study, plasma-soluble urokinase plasminogen activator receptor (suPAR) levels played a role in the decline in renal function in cohorts with APOL1 variant milieus [57]. In these studies, APOL1G1/G2-related risk was reduced in patients with lower suPAR, but enhanced in those with higher suPAR levels. It was suggested that APOL1 protein variants (G1 and G2) had higher affinity for suPAR-activated α_v_β_3_ integrin than APOL1 G0. On this account, APOL1 variants augmented α_v_β_3_ integrin activation, leading to a podocyte injury. The latter led to proteinuria in mice in a suPAR-dependent manner. Interestingly, Vitamin D attenuated the podocyte expression of Urokinase plasminogen activator receptor (uPAR) and decreased the elevated circulating suPAR levels associated with systemic inflammation [58]. Because Vitamin D enhances the transcription of VDR and works through VDR [59], it may be likely that VDR indirectly modulates the suPAR levels. Since miR193a downregulated VDR, it may also increase blood suPAR levels. It would be worth investigating this aspect in future studies.

### Strengths and Limitations

The present hypothesis provides a basis for supporting the role of APOL1 and its variants in activating the RAS in podocytes. It also delineates the mechanistic role of the APOL1-miR193a axis in activating the RAS and the involvement of the WT1-BASP1 complex. This study also provides data on the occurrence of the activation of the RAS in a classical mouse model of FSGS-miR193aTr mice. However, the present study was not designed to validate a causal role of RAS in the development and progression of APOL1G1/G2 mediated podocyte injury or the development of FSGS. Therefore, the present study’s strength is limited to exploring the role of APOL1 dynamics in the modulation of the RAS in kidneys, but a cause-and-effect relationship of the RAS in APOL1-mediated FSGS has yet to be established. Since controlling podocyte variant protein expression levels is challenging, we used immortalized cell lines expressing stable APOL1G0, G1, and G2 to minimize the disparity. It is important to note that, while overexpression studies have limitations, they still provide valuable insights into the potential effects of APOL1 variants on RAS activation. A thorough analysis of VDR, RAS elements, and miR193a and APOL1 in healthy human and FSGS biopsies will be needed in order to validate our findings in future studies.

## 5. Conclusions

The enhanced expression of the APOL1 non-risk allele (G0) escalated VDR expression through a reduction in miR193a and resulted in a lack of activation of the RAS, contributing to podocyte health preservation. In contrast, the expression of APOL1 risk alleles (G1 and G2) escalated miR193a levels, and lowering of VDR expression led to the activation of the RAS, which contributed to the health of the compromised podocytes and to glomerular injury.

## Figures and Tables

**Table 1:** Primer sequences used to amplify specific mRNAs.

Gene	
mNphs1-F	GTGCCCTGAAGGACCCTACT
mNphs1-R	CCTGTGGATCCCTTTGACAT
mWt1-F	GAGAGCCAGCCTACCATCC
mWt1-R	GGGTCCTCGTGTTTGAAGGAA
mRenin-F	TGAGCATCAGCAAGACTGACTCC
mRenin-R	AGGATGAACCAGTGTCCACCACTAC
mAce1-F	CCACTATGGGTCCGAGTACATCAA
mAce-R	AGGGCGCCACCAAATCATAG
mAgt-F	CTGGATTTATCCACTGACCCAGTTC
mAgt-R	TGGACTCCAGGCAGCTGAGA
mAgtr1a-F	TGGGCGTCATCCATGACTGTA
mAgtr1a-R	TGAGTGCGACTTGGCCTTTG
mAgtr1b-F	CTGCTATGCCCATCACCATCTG
mAgtr1b-R	GATAACCCTGCATGCGACCTG
mAgtr2-F	GTGCATGCGGGAGCTGAGTA
mAgtr2-R	ATTGGTGCCAGTTGCGTTGA
*mVDR*-F	CACCTGGCTGATCTTGTCAGT
*mVDR*-R	CTGGTCATCAGAGGTGAGGTC
*hVDR*-F	CCTTCACCATGGACGACATG
*hVDR*-R	CGGCTTTGGTCACGTCACT
mCd2ap-F	AGCGAATCAGCACTTATGGA
mCd2ap-R	CCACCAGCCTTCTTCTACCT
mPodx-F	CCTGAACCTCACAGGAAACACC
mPodx-R	TGGAACAGATGCCAGCCGTATG

## Data Availability

The original contributions presented in the study are included in the article, further inquiries can be directed to the corresponding author.
